# Chemical Composition and Porosity Characteristics of Various Calcium Silicate-Based Endodontic Cements

**DOI:** 10.1155/2018/2784632

**Published:** 2018-02-01

**Authors:** Seok Woo Chang

**Affiliations:** Department of Conservative Dentistry, School of Dentistry, Kyung Hee University, Seoul, Republic of Korea

## Abstract

Chemical composition and porosity characteristics of calcium silicate-based endodontic cements are important determinants of their clinical performance. Therefore, the aim of this study was to investigate the chemical composition and porosity characteristics of various calcium silicate-based endodontic cements: MTA-angelus, Bioaggregate, Biodentine, Micromega MTA, Ortho MTA, and ProRoot MTA. The specific surface area, pore volume, and pore diameter were measured by the porosimetry analysis of N2 adsorption/desorption isotherms. Chemical composition and powder analysis by scanning electron microscope (SEM) and energy dispersive spectroscopy (EDS) were also carried out on these endodontic cements. Biodentine and MTA-angelus showed the smallest pore volume and pore diameter, respectively. Specific surface area was the largest in MTA-angelus. SEM and EDS analysis showed that Bioaggregate and Biodentine contained homogenous, round and small particles, which did not contain bismuth oxide.

## 1. Introduction

Mineral trioxide aggregate (MTA) was introduced in endodontic field as root end filling material and perforation repair material in early 1990s [1]. Due to its superior biocompatibility [2] and sealing ability [3], MTA has been widely used for perforation repair [4], root end filling [5], pulp capping [6], one-visit apexification [7], and pulpal revascularization [8]. However, MTA has been described to have drawbacks such as long setting time [9], tooth discoloration potential [10], and handling difficulty [11]. To overcome these drawbacks, many calcium silicate-based cements such as MTA-angelus [12], Bioaggregate [13], Biodentine [12], Micromega MTA (MM-MTA) [6], and Ortho MTA [14] have been introduced in market and showed good clinical and experimental results.

There are many reports that proved superior sealing ability of MTA in the MTA-tooth interface [15, 16]. However, the porosity existing in MTA itself has not been studied extensively [17–19]. Considering that the porosity of MTA is related to its ability to resist microbial penetration and leakage [20], there is relative lack of knowledge on this issue currently.

Thus, the aim of this study was to investigate the pore volume, pore diameter, and the specific surface area of various commercial calcium silicate-based endodontic cements. The surface morphology and chemical compositions of these cements were also investigated.

## 2. Materials and Methods

### 2.1. Materials Used

The materials used in this study were MTA-angelus, Bioaggregate, Biodentine, MM-MTA, Ortho MTA, and ProRoot MTA. The compositions of these materials are listed in Table 1.

### 2.2. BET Surface Area and Porosimetry Analyzer

Surface area and pore structure were measured by N2 adsorption/desorption isotherms (ASAP 2020 series) at 77 and 273 K for nitrogen and carbon dioxide within relative pressures from 0 to 1.0 and from 0 to 0.03, respectively. Before analysis, the samples were degassed in the degas port of the adsorption analyzer at 423 K for 10 hours. The surface area, pore volume, and pore diameter were analyzed using ASAP 2020 v3.00 software (Micromeritics, Norcross, GA, USA).

### 2.3. Scanning Electron Microscope (SEM) and Energy Dispersive Spectroscopy (EDS) Analysis

The morphology of the powders and chemical constitutions was measured on JEOL JSM-6700 scanning electron microscope. Prior to SEM measurement, the samples were coated with platinum using sputter for 45 seconds.

## 3. Results

### 3.1. BET Surface Area and Porosimetry Analysis

The specific surface area (m^2^/g), pore volume (cm^3^/g), and pore diameter (nm) values of all the samples are listed in Table 2. Specific surface area was the largest in MTA-angelus and the smallest in ProRoot MTA. Pore volume was the largest in MTA-angelus and the smallest in Biodentine. Pore diameter was the largest in MM-MTA and the smallest in MTA-angelus.

### 3.2. Scanning Electron Microscope (SEM) and Energy Dispersive Spectroscopy (EDS) Analysis

**Figure 1:**
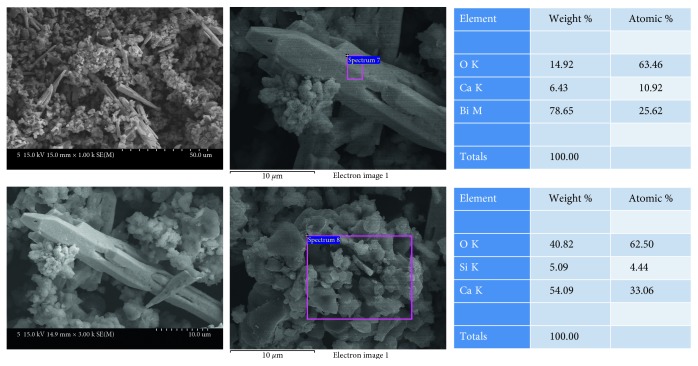
SEM and EDS analysis results of MTA-angelus.

MTA-angelus (Figure 1) showed multiple aggregates of round particles. EDS analysis showed that these round particles are mainly composed of calcium and silica. Among these round particles, long spindle-shaped particles were shown. EDS analysis showed that these long spindle-shaped particles were mainly composed of bismuth.

**Figure 2:**
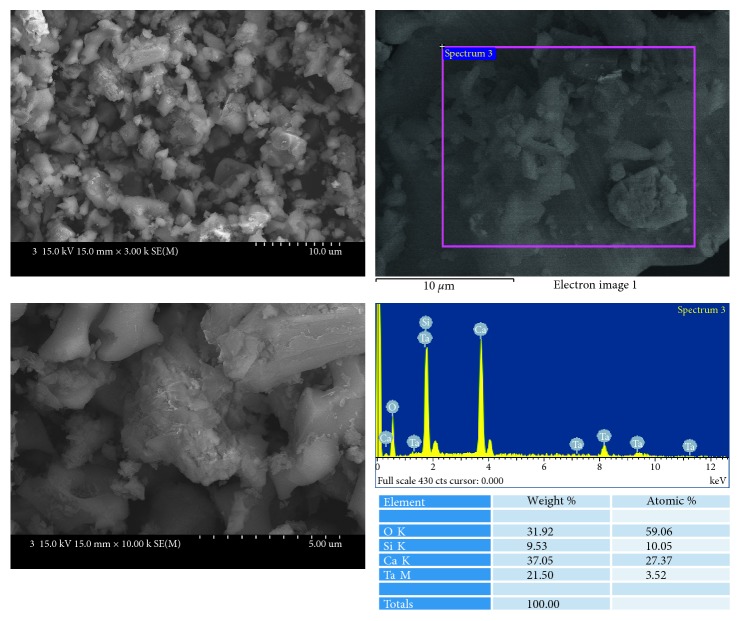
SEM and EDS analysis results of Bioaggregate.

Bioaggregate (Figure 2) showed relatively homogenous aggregates of small round particles. EDS analysis showed that these particles were mainly composed of calcium, silicon, and tantalum. Bioaggregate did not contain bismuth.

**Figure 3:**
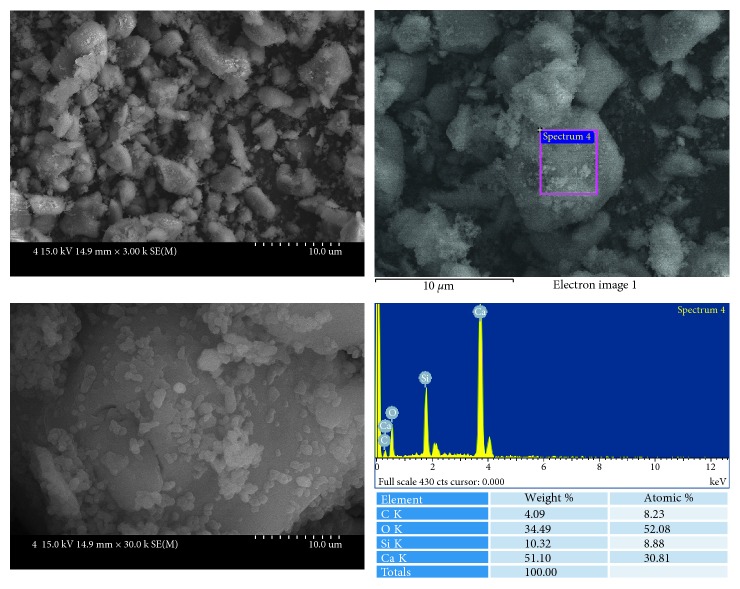
SEM and EDS analysis results of Biodentine.

Biodentine (Figure 3) showed that relatively large particles were covered with small particles. EDS analysis showed that these particles were mainly composed of calcium and silicon.

**Figure 4:**
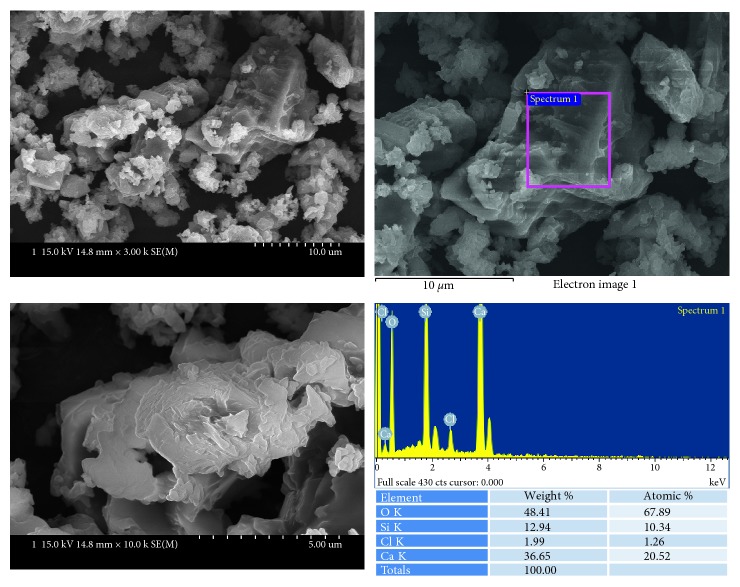
SEM and EDS analysis results of MM-MTA.

MM-MTA (Figure 4) also showed the mixtures of relatively larger particles and smaller particles. EDS analysis showed that these particles were mainly composed of calcium and silicon.

**Figure 5:**
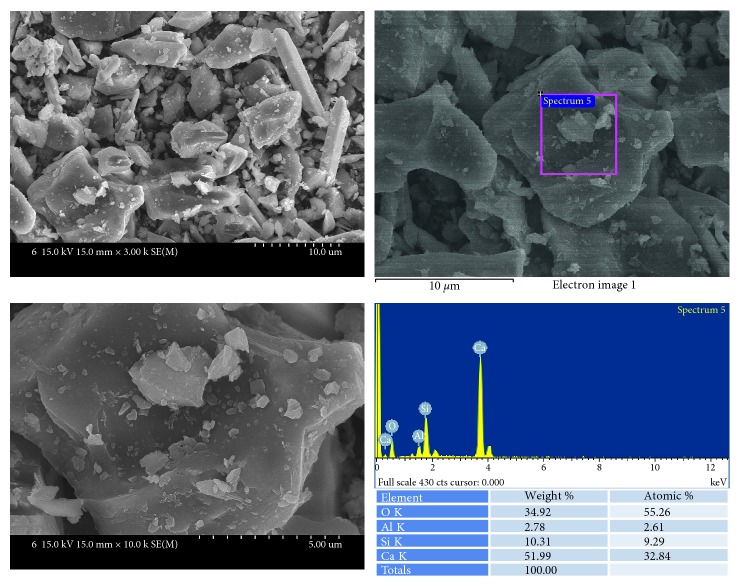
SEM and EDS analysis results of Ortho MTA.

Ortho MTA (Figure 5) showed large particles, small particles, and long spindle-shaped particles at the same time. All these particles were shown to be mainly composed of calcium and silicon.

**Figure 6:**
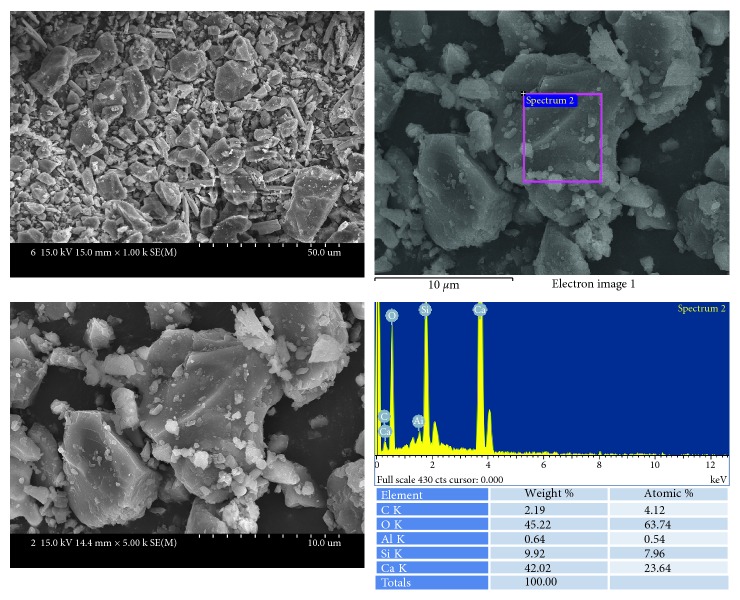
SEM and EDS analysis results of ProRoot MTA.

ProRoot MTA (Figure 6) showed relatively homogenous particles which are mainly composed of calcium and silicon.

## 4. Discussion

Porosity of mineral trioxide aggregate is important in that it is related to bacterial leakage [20]. However, there are few studies which investigated the porosity of MTA [17–19]. Regarding the porosity characteristics, one previous study [17] reported that the apparent porosity of ProRoot MTA was 29.36% while that of Dycal was 9.04%. However, this study used Archimedes' principle to calculate the porosity of MTA samples. In this reason, this study had a limitation that it could not give information regarding the characteristics such as pore diameter and specific surface area of MTA.

Porosity-related properties of a certain material are specific surface area (m^2^/g), pore volume (cm^3^/g), and pore diameter [22]. Most previous studies which investigated MTA porosity used mercury intrusion porosimetry [18, 19]. It was reported that the detection range of mercury intrusion porosimetry is from 3 nm to 200 *μ*m, whereas that of N2 adsorption/desorption isotherms is from 0.3 nm to 300 nm [22]. According to this report [22], N2 adsorption/desorption isotherms can detect the small pores which could not be detected by mercury intrusion porosimetry. In this reason, the study of porosity of MTA using N2 adsorption/desorption isotherms as well as mercury intrusion porosimetry could be regarded as ideal methods.

The previous studies reported that the pore volume for ProRoot MTA was 0.1025 cm^3^/g at pH 7.4 [19]. The pore volume for ProRoot MTA was 0.0097 cm^3^/g in this study. This difference could be attributed to the experimental conditions such as time elapsed for MTA setting and the environment around the MTA setting.

The pore volume inside the specimen was the largest in MTA-angelus group (0.016 cm^3^/g). The pore volume inside Bioaggregate and Ortho MTA was the same and was 0.014 cm^3^/g. The pore volume of MM-MTA was 0.0086 cm^3^/g. The pore volume of Biodentine was the smallest of all the tested groups. (0.0080 cm^3^/g).

In addition to the total pore volume, the size of pore diameter is important [19]. Unfortunately, there has been no study which evaluated pore diameters of mineral trioxide aggregate. In the present study, pore diameter was the largest in MM-MTA (21.5 nm) and decreased in the order of ProRoot MTA, Biodentine, Bioaggregate, Ortho MTA, and MTA-angelus. MTA-angelus has the smallest pore diameter, and it was 9.3 nm. Considering that the average size of *Enterococcus faecalis* (representative endodontic bacterium) is 0.6–2.5 *μ*m [23], it is quite unlikely that bacteria could penetrate well-condensed and hydrated MTA. Another characteristic investigated in this study was specific surface area. Specific surface area could affect the adhesion of contacting cells [24]. The larger surface area is considered to be the more favorable condition to cellular adhesion [24]. In the present study, the specific surface area was the largest in MTA-angelus and decreased in the order of Bioaggregate, Ortho MTA, Biodentine, MM-MTA, and ProRoot MTA. ProRoot MTA has the smallest specific surface area, and it was 3.2 m^2^/g. The effect of these different specific surface areas should be investigated further in future study.

## 5. Conclusion

In conclusion, this study showed that Biodentine and MTA-angelus showed the smallest pore volume and pore diameter, respectively, which could be regarded as superior physicochemical properties from the perspective of clinical endodontics.

## Figures and Tables

**Table 1:** Names and compositions of calcium silicate-based endodontic cements which were used in this study.

Products	Compositions
MTA-angelus (Londrina, PR, Brazil)	Tricalcium silicate, dicalcium silicate, tricalcium aluminate, tetracalcium aluminoferrite, bismuth oxide (MSDS)
Bioaggregate (Diadent, Burnaby, Canada)	Tricalcium silicate, dicalcium silicate, tantalum pentoxide, calcium phosphate monobasic, amorphous silicon oxide (MSDS)
Biodentine (Septodont, St. Maur-des-Fossés, France)	Tricalcium silicate, dicalcium silicate, calcium carbonate and oxide, iron oxide, zirconium oxide [21]
MM-MTA (MicroMega, Besançon, France)	Mixture of several mineral oxides and bismuth oxides (MSDS)
Ortho MTA (BioMTA, Seoul, Korea)	Calcium carbonate, silicon dioxide, aluminum oxide, dibismuth trioxide (MSDS)
ProRoot MTA (Dentsply, Tulsa, OK, USA)	Portland cement, bismuth oxide (MSDS)

**Table 2:** Porosity in tested calcium silicate-based endodontic cements.

	MTA-angelus	Bioaggregate	Biodentine	MM-MTA	Ortho MTA	ProRoot MTA
*S*_*A*_	6.2	5.5	4.0	3.5	4.5	3.2
*V*_pore_	0.016	0.014	0.0080	0.0086	0.014	0.0097
*d*_pore_	9.3	11.9	13.5	21.5	11.1	17.3

*S*_*A*_: specific surface area (m^2^/g) calculated by BET equation; *V*_pore_: pore volume (cm^3^/g) calculated by BJH equation; *d*_pore_: pore diameter (nm) calculated by BJH equation.
